# Supplementary material for: Functional Characterization of ShK Domain-Containing Protein in the Plant-Parasitic Nematode Bursaphelenchus xylophilus
Source: Plants (Basel). 2024 Jan 30;13(3):404. doi: 10.3390/plants13030404 (PMC10857297; doi:10.3390/plants13030404)
Supplement: Supplementary file 1 [file plants-13-00404-s001.zip › plants-2784445-supplementary.pdf]

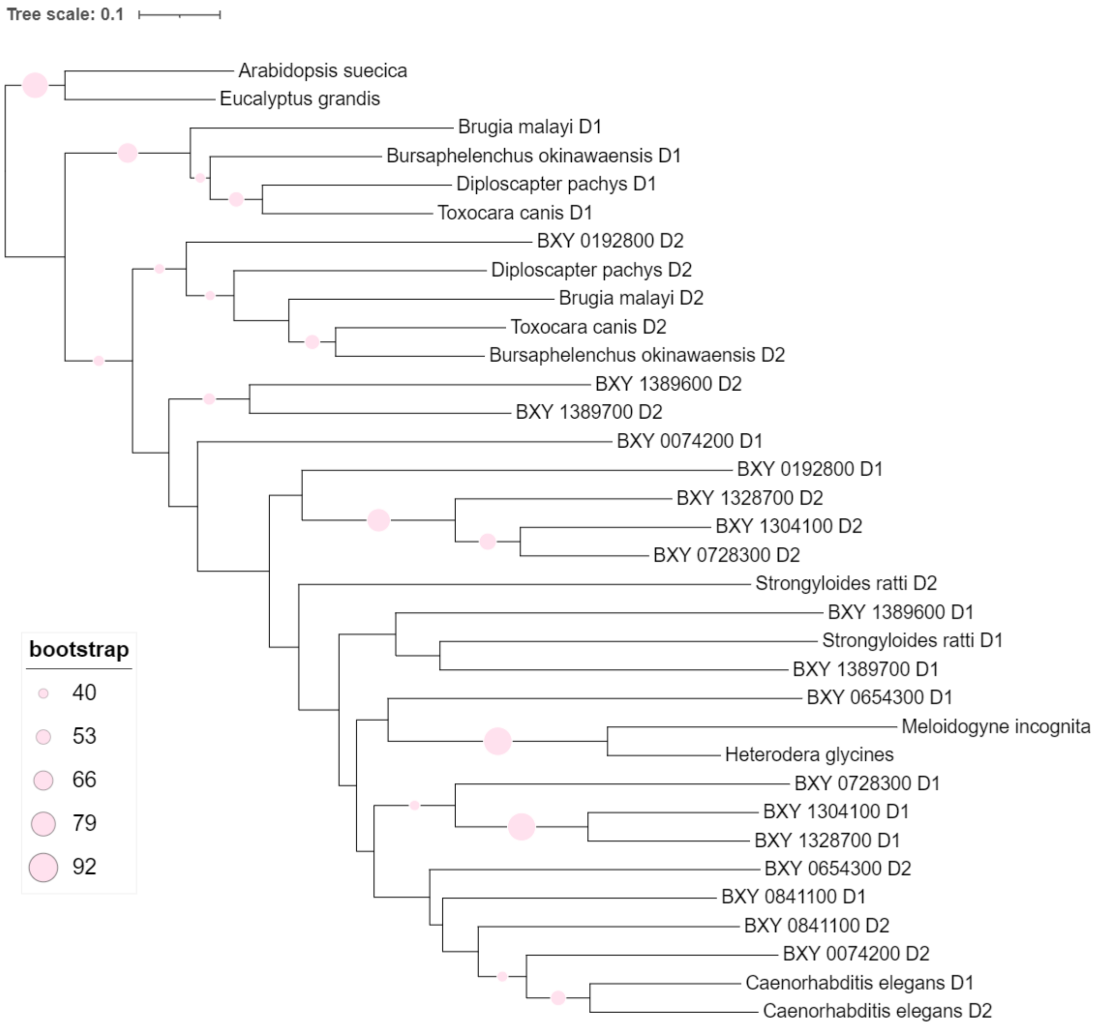

Figure S1: Neighbor-joining (NJ) phylogeny tree that represents the protein sequence similarity between of the nine candidate genes potential specific *B. xylophilus* predicted ShK domain-containing proteins and other organisms, such as plant-parasitic, animal-parasitic and free-living nematodes and plants also ShK domain-containing proteins with domain D1 and domain D2.
